# Supplementary material for: Modelling Skylarks (Alauda arvensis) to Predict Impacts of Changes in Land Management and Policy: Development and Testing of an Agent-Based Model
Source: PLoS One. 2013 Jun 6;8(6):e65803. doi: 10.1371/journal.pone.0065803 (PMC3675089; doi:10.1371/journal.pone.0065803)
Supplement: Supporting Information S4 — The skylark ODdox as a zipped archive. (ZIP) [file pone.0065803.s004.zip › Skylark_ODdox/map__cfg_8cpp.html]

ALMaSS Skylark ODdox: map\_cfg.cpp File Reference


ALMaSS Skylark ODdox
 2.0


- Main Page
- Related Pages
- Classes
- Files

- File List
- File Members

Variables

map\_cfg.cpp File Reference

`#include "configurator.h"`  
`#include "tov_declaration.h"`

|  |  |
| --- | --- |
| Variables | |
| CfgBool | g\_farm\_enable\_crop\_rotation ("FARM\_ENABLE\_CROP\_ROTATION", CFG\_CUSTOM, true) |
| CfgBool | g\_farm\_fixed\_crop\_enable ("FARM\_FIXED\_CROP\_ENABLE", CFG\_CUSTOM, false) |
| CfgInt | g\_farm\_fixed\_crop\_type ("FARM\_FIXED\_CROP\_TYPE", CFG\_CUSTOM, 22) |
| CfgBool | g\_farm\_fixed\_rotation\_enable ("FARM\_FIXED\_ROTATION\_ENABLE", CFG\_CUSTOM, false) |
| CfgBool | g\_farm\_fixed\_rotation\_farms\_async ("FARM\_FIXED\_ROTATION\_FARMS\_ASYNC", CFG\_CUSTOM, false) |
| CfgInt | g\_farm\_fixed\_rotation\_farmtype ("FARM\_FIXED\_ROTATION\_FARMTYPE", CFG\_CUSTOM, 0) |
| CfgInt | g\_farm\_test\_crop\_daystorun ("FARM\_TEST\_CROP\_DAYSTORUN", CFG\_CUSTOM, 36500) |
| CfgInt | g\_farm\_test\_crop\_farmtype ("FARM\_TEST\_CROP\_FARMTYPE", CFG\_CUSTOM, 5) |
| CfgStr | g\_farm\_test\_crop\_filename ("FARM\_TEST\_CROP\_FILENAME", CFG\_CUSTOM,"OWinterRape\_plant\_1.txt") |
| CfgBool | g\_farm\_test\_crop\_reportdaily ("FARM\_TEST\_CROP\_REPORTDAILY", CFG\_CUSTOM, false) |
| CfgInt | g\_farm\_test\_crop\_sample\_x ("FARM\_TEST\_CROP\_SAMPLE\_X", CFG\_CUSTOM, 1328) |
| CfgInt | g\_farm\_test\_crop\_sample\_y ("FARM\_TEST\_CROP\_SAMPLE\_Y", CFG\_CUSTOM, 1328) |
| CfgInt | g\_farm\_test\_crop\_type ("FARM\_TEST\_CROP\_TYPE", CFG\_CUSTOM, 22) |
| CfgInt | g\_map\_le\_border\_chance ("MAP\_LE\_BORDER\_CHANCE", CFG\_CUSTOM, 25) |
| CfgBool | g\_map\_le\_borders ("MAP\_LE\_BORDERS", CFG\_CUSTOM, false) |
| CfgBool | g\_map\_le\_unsprayedmargins ("MAP\_LE\_UNSPRAYEDMARGINS", CFG\_CUSTOM, false) |
| CfgInt | g\_map\_le\_unsprayedmargins\_chance ("MAP\_LE\_UMARGIN\_CHANCE", CFG\_CUSTOM, 25) |
| CfgInt | g\_map\_maxpolyref ("MAP\_MAX\_POLYREF", CFG\_PUBLIC, 200000) |
| CfgBool | g\_map\_orchards\_borders ("MAP\_ORCHARDS\_BORDERS", CFG\_CUSTOM, false) |
| CfgInt | g\_map\_orchardsborderwidth ("MAP\_ORCHARDS\_BORDER\_WIDTH", CFG\_CUSTOM, 5) |

---

## Variable Documentation

CfgBool g\_farm\_enable\_crop\_rotation("FARM\_ENABLE\_CROP\_ROTATION", CFG\_CUSTOM, true)

Referenced by Farm::GetNextCropIndex().

CfgBool g\_farm\_fixed\_crop\_enable("FARM\_FIXED\_CROP\_ENABLE", CFG\_CUSTOM, false)

Referenced by Farm::GetNextCropStartDate(), Farm::HandleEvents(), and Farm::InitiateManagement().

CfgInt g\_farm\_fixed\_crop\_type("FARM\_FIXED\_CROP\_TYPE", CFG\_CUSTOM, 22)

Referenced by Farm::HandleEvents(), and Farm::InitiateManagement().

CfgBool g\_farm\_fixed\_rotation\_enable("FARM\_FIXED\_ROTATION\_ENABLE", CFG\_CUSTOM, false)

Referenced by FarmManager::CreateFarms(), and Farm::GetFirstCropIndex().

CfgBool g\_farm\_fixed\_rotation\_farms\_async("FARM\_FIXED\_ROTATION\_FARMS\_ASYNC", CFG\_CUSTOM, false)

Referenced by Farm::GetFirstCropIndex().

CfgInt g\_farm\_fixed\_rotation\_farmtype("FARM\_FIXED\_ROTATION\_FARMTYPE", CFG\_CUSTOM, 0)

Referenced by FarmManager::CreateFarms().

CfgInt g\_farm\_test\_crop\_daystorun("FARM\_TEST\_CROP\_DAYSTORUN", CFG\_CUSTOM, 36500)

CfgInt g\_farm\_test\_crop\_farmtype("FARM\_TEST\_CROP\_FARMTYPE", CFG\_CUSTOM, 5)

CfgStr g\_farm\_test\_crop\_filename("FARM\_TEST\_CROP\_FILENAME", CFG\_CUSTOM,"OWinterRape\_plant\_1.txt")

CfgBool g\_farm\_test\_crop\_reportdaily("FARM\_TEST\_CROP\_REPORTDAILY", CFG\_CUSTOM, false)

CfgInt g\_farm\_test\_crop\_sample\_x("FARM\_TEST\_CROP\_SAMPLE\_X", CFG\_CUSTOM, 1328)

CfgInt g\_farm\_test\_crop\_sample\_y("FARM\_TEST\_CROP\_SAMPLE\_Y", CFG\_CUSTOM, 1328)

CfgInt g\_farm\_test\_crop\_type("FARM\_TEST\_CROP\_TYPE", CFG\_CUSTOM, 22)

CfgInt g\_map\_le\_border\_chance("MAP\_LE\_BORDER\_CHANCE", CFG\_CUSTOM, 25)

Percentile chance of a field getting a border, if MAP\_LE\_BORDERS is enabled above.

Referenced by Landscape::ReadPolys().

CfgBool g\_map\_le\_borders("MAP\_LE\_BORDERS", CFG\_CUSTOM, false)

If true will make and use new borders around certain types of farmed areas. The area used is 'stolen' from a parent landscape element.

Referenced by Landscape::Landscape(), and Landscape::ReadPolys().

CfgBool g\_map\_le\_unsprayedmargins("MAP\_LE\_UNSPRAYEDMARGINS", CFG\_CUSTOM, false)

Referenced by Landscape::Landscape(), and Landscape::ReadPolys().

CfgInt g\_map\_le\_unsprayedmargins\_chance("MAP\_LE\_UMARGIN\_CHANCE", CFG\_CUSTOM, 25)

Referenced by Landscape::ReadPolys().

CfgInt g\_map\_maxpolyref("MAP\_MAX\_POLYREF", CFG\_PUBLIC, 200000)

Referenced by Landscape::Landscape(), and Landscape::ReadPolys().

CfgBool g\_map\_orchards\_borders("MAP\_ORCHARDS\_BORDERS", CFG\_CUSTOM, false)

If true will make and use new borders around tole\_orchard areas. The area used is 'stolen' from a parent landscape element.

Referenced by Landscape::Landscape().

CfgInt g\_map\_orchardsborderwidth("MAP\_ORCHARDS\_BORDER\_WIDTH", CFG\_CUSTOM, 5)

The width of the orchard borders if any

Referenced by Landscape::Landscape().


- CJT
- MSVC
- ALMaSS Working Source
- Landscape
- map\_cfg.cpp
- Generated on Thu Jan 10 2013 13:15:35 for ALMaSS Skylark ODdox by
   1.8.1.1
